# Resection of cerebellar tumours causes widespread and functionally relevant white matter impairments

**DOI:** 10.1002/hbm.25317

**Published:** 2021-01-07

**Authors:** Carlos Alexandre Gomes, Katharina M. Steiner, Nicolas Ludolph, Tamas Spisak, Thomas M. Ernst, Oliver Mueller, Sophia L. Göricke, Franziska Labrenz, Winfried Ilg, Nikolai Axmacher, Dagmar Timmann

**Affiliations:** ^1^ Department of Neuropsychology Institute of Cognitive Neuroscience, Faculty of Psychology, Ruhr University Bochum Bochum Germany; ^2^ Department of Neurology Essen University Hospital, University of Duisburg‐Essen Essen Germany; ^3^ Cognitive Neurology, Section Computational Sensomotorics Hertie Institute for Clinical Brain Research and Center for Integrative Neuroscience (HIH), Eberhard Karls University Tübingen Germany; ^4^ Predictive Neuroimaging Lab Institute for Artificial Intelligence in Medicine ‐ Institute of Diagnostic and Interventional Radiology and Neuroradiology, Essen University Hospital Essen Germany; ^5^ Department of Neurosurgery Klinikum Dortmund Dortmund Germany; ^6^ Department of Neurosurgery Essen University Hospital, University of Duisburg‐Essen Essen Germany; ^7^ Institute of Diagnostic and Interventional Radiology and Neuroradiology Essen University Hospital, University of Duisburg‐Essen Essen Germany

**Keywords:** cerebellum, diffusion tensor imaging, eyeblink conditioning, lesion‐symptom mapping, motor skill learning

## Abstract

Several diffusion tensor imaging studies reveal that white matter (WM) lesions are common in children suffering from benign cerebellar tumours who are treated with surgery only. The clinical implications of WM alterations that occur as a direct consequence of cerebellar disease have not been thoroughly studied. Here, we analysed structural and diffusion imaging data from cerebellar patients with chronic surgical lesions after resection for benign cerebellar tumours. We aimed to elucidate the impact of focal lesions of the cerebellum on WM integrity across the entire brain, and to investigate whether WM deficits were associated with behavioural impairment in three different motor tasks. Lesion symptom mapping analysis suggested that lesions in critical cerebellar regions were related to deficits in savings during an eyeblink conditioning task, as well as to deficits in motor action timing. Diffusion imaging analysis of cerebellar WM indicated that better behavioural performance was associated with higher fractional anisotropy (FA) in the superior cerebellar peduncle, cerebellum's main outflow path. Moreover, voxel‐wise analysis revealed a global pattern of WM deficits in patients within many cerebral WM tracts critical for motor and non‐motor function. Finally, we observed a positive correlation between FA and savings within cerebello‐thalamo‐cortical pathways in patients but not in controls, showing that saving effects partly depend on extracerebellar areas, and may be recruited for compensation. These results confirm that the cerebellum has extended connections with many cerebral areas involved in motor/cognitive functions, and the observed WM changes likely contribute to long‐term clinical deficits of posterior fossa tumour survivors.

## Graphical abstract

We investigated the impact of focal lesions of the cerebellum on whole‐brain white matter integrity and their relationship with behavioural impairments in three different motor tasks. Better behavioural performance was linked to higher fractional anisotropy in the superior cerebellar peduncle. We also observed widespread white matter deficits in patients relative to controls within many cerebral white matter tracts critical for motor and non‐motor function.

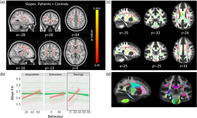

## 1 INTRODUCTION

Cerebellar tumours are very common in childhood (Brandão & Young Poussaint, 2017), with medulloblastomas and pilocytic astrocytomas being two of the most prevalent. Medulloblastomas are cancerous so children need to receive adjuvant chemo‐ and radiotherapy. Detrimental effects of radiotherapy on cerebral white matter and accompanying intellectual deficits in children treated for medulloblastomas are well‐documented (Chevignard, Câmara‐Costa, Doz, & Dellatolas, 2017; Dennis, Spiegler, Ross Hetherington, & Greenberg, 1996; Radcliffe, Bunin, Sutton, Goldwein, & Phillips, 1994). Studies using advanced neuroimaging techniques, such as diffusion tensor imaging (DTI), revealed that white matter lesions are also common in children suffering from benign pilocytic astrocytomas who are treated with surgery only (Rueckriegel, Bruhn, Thomale, & Hernáiz Driever, 2015). The clinical implications of white matter alterations that occur as a direct consequence of cerebellar disease have not been studied in detail. Most studies were interested in predictors of the posterior fossa or cerebellar mutism syndrome which appear to be correlated with lesions of the superior cerebellar peduncles (Ojemann et al., 2013). A few studies reported that white matter alterations in children treated for benign cerebellar tumours correlated with motor and cognitive deficits assessed by clinical ataxia rating scales, simple motor tasks and standard cognitive tests (Law et al., 2011; Oh et al., 2017; Palmer et al., 2012; Rueckriegel et al., 2015). More detailed analyses of cerebellar‐dependent motor dysfunction are lacking.

Cerebellar disorders result in well‐known deficits of motor coordination, motor learning and motor timing (Bodranghien et al., 2016; Diedrichsen & Bastian, 2014; Morton & Bastian, 2004). The underlying cause is thought to be the inability to predict the sensory consequences of movements (Mauk, Li, Khilkevich, & Halverson, 2014; Mauk, Medina, Nores, & Ohyama, 2000; Miall & Wolpert, 1996; Synofzik, Lindner, & Thier, 2008; Tseng, Diedrichsen, Krakauer, Shadmehr, & Bastian, 2007). In the present study, two motor learning tasks and one motor performance task were applied, all of which required predictive timing of motor action. The first task was a classical eyeblink conditioning paradigm (Ernst et al., 2016; Gerwig et al., 2003; Hesslow, Jirenhed, Rasmussen, & Johansson, 2013). In this task, participants learn that an initially neutral conditioned stimulus (CS), frequently a tone, is followed by an aversive unconditioned stimulus (US), most commonly an air puff directed to the eye, and learn to close their eyes in a precisely timed manner after the onset of the CS and prior to occurrence of the US (Gerwig et al., 2005; Woodruff‐Pak, 1997). The second task was a ‘metronome’ task, in which participants had to perform movements in synchrony with a predictable visual stimulus to test their basic predictive action timing capabilities (Ludolph et al., n.d.). The third task was a virtual cart‐pole balancing task, in which participants learn to control a virtual simulation of the cart‐pole system, a task which requires precisely timed motor action by predicting complex object dynamics (Ludolph et al., n.d. bioRxiv). Thus, all three tasks include specific requirements on action timing with increasing complexity for the different tasks.

The aim of the present study was to test for possible correlations between motor learning and timing deficits, and to investigate white matter changes in a group of young adults with chronic surgical lesions for benign cerebellar tumours. Behavioural data of the two learning tasks have been published elsewhere (Ernst et al., 2016; Ludolph et al., n.d. bioRxiv). As expected, the participants with chronic cerebellar lesions were impaired in the acquisition of conditioned eyeblink responses (Ernst et al., 2016), and in learning of the virtual cart‐pole task (Ludolph et al., n.d. bioRxiv). Voxel‐based lesion symptom mapping (VBLSM) had also been performed but was limited to the surgical lesion within the cerebellum and to descriptive and binomial statistical methods. In the present study, DTI was used to assess damage to cerebellar outflow tracts, particularly the superior cerebellar peduncle and cerebello‐thalamo‐cortical white matter tracts, which was complemented by more advanced statistical methods of VBLSM, and correlated with motor learning and predictive timing deficits. This is of interest because not only does it allow for more detailed predictions of motor outcome in children treated for benign cerebellar tumours, but also widens our understanding of the contribution of the cerebello‐cortical network to motor control.

## 2 METHODS

### 2.1 Participants

Fifty‐three participants (mean age = 25.39, *SD* = 5.27, range 17–39 years, 32 females) took part in this study and gave fully informed consent. Of these 53 subjects, 19 had chronic surgical lesions of the cerebellum for treatment of benign cerebellar tumours, while the remaining 34 participants were healthy controls. Twelve participants were treated for astrocytoma WHO grade I, two for astrocytoma WHO grade II, four for vascular tumours and one for a dermoid cyst. Mean time since surgery was 11.3 years (*SD* = 5.7), and age at diagnosis was 14.6 years (*SD* = 7.5 years). All patients had only had surgery once for their tumour and none showed postoperative cerebellar mutism. In addition, all patients received in‐patient rehabilitation, which lasted, on average, 2–3 months. Sixteen cerebellar participants were right‐handed and one participant was left‐handed based on the Edinburgh handedness inventory (Oldfield, 1971). Two right‐handed cerebellar participants were trained to be left‐handed after surgery. The database of the local neurosurgery department was used for the recruitment of cerebellar participants. The inclusion criteria were that the tumours needed to be benign (e.g., no medulloblastomas) and that no adjuvant chemo‐ or radiotherapy had been applied. In one cerebellar participant tested, MR images could not be acquired because of a recently implanted contraceptive intrauterine device which was not MRI safe. Thus, our final cerebellar participant sample consisted of 18 patients. At the time of the surgery, 15 of the 18 patients showed signs of increased intracranial pressure in brain MRI: one required a permanent shunt, the remaining 14 required an external ventricle drainage, which is temporary, and was removed within 3–5 days.

Thirty‐four healthy control participants with no history of any neurological disease were also recruited. For the analysis of eyeblink conditioning data, we selected 18 matched healthy participants from the 34 controls (mean age 25.5, *SD* 4.9 years; range 18–38 years; 12 female; one left‐handed). All participants were tested at the Department of Neurology in Essen, and also received brain MRI scans.

For the ‘metronome’ task and the cart‐pole balancing task 19 sex‐ and age‐matched self‐reportedly neurologically healthy control subjects participated in the experiment. These participants were tested at the HIH in Tübingen. In this control group, no brain MRI scans were available.

All cerebellar participants were examined by an experienced neurologist during the day of the experiments. Clinical ataxia scores were acquired using the International Cooperative Ataxia Rating Scale (ICARS; Trouillas et al., 1997). For a more detailed description of these participants see Ernst et al. (2016) and Ludolph et al., n.d. (bioRxiv). This study was approved by the local ethics committee.

### 2.2 Behavioural paradigms

Behavioural data in the two motor learning tasks have been published elsewhere (Ernst et al., 2016; Ludolph et al., n.d. bioRxiv). The paradigms are briefly summarised below.

#### 2.2.1 Eyeblink conditioning

**FIGURE 1:**
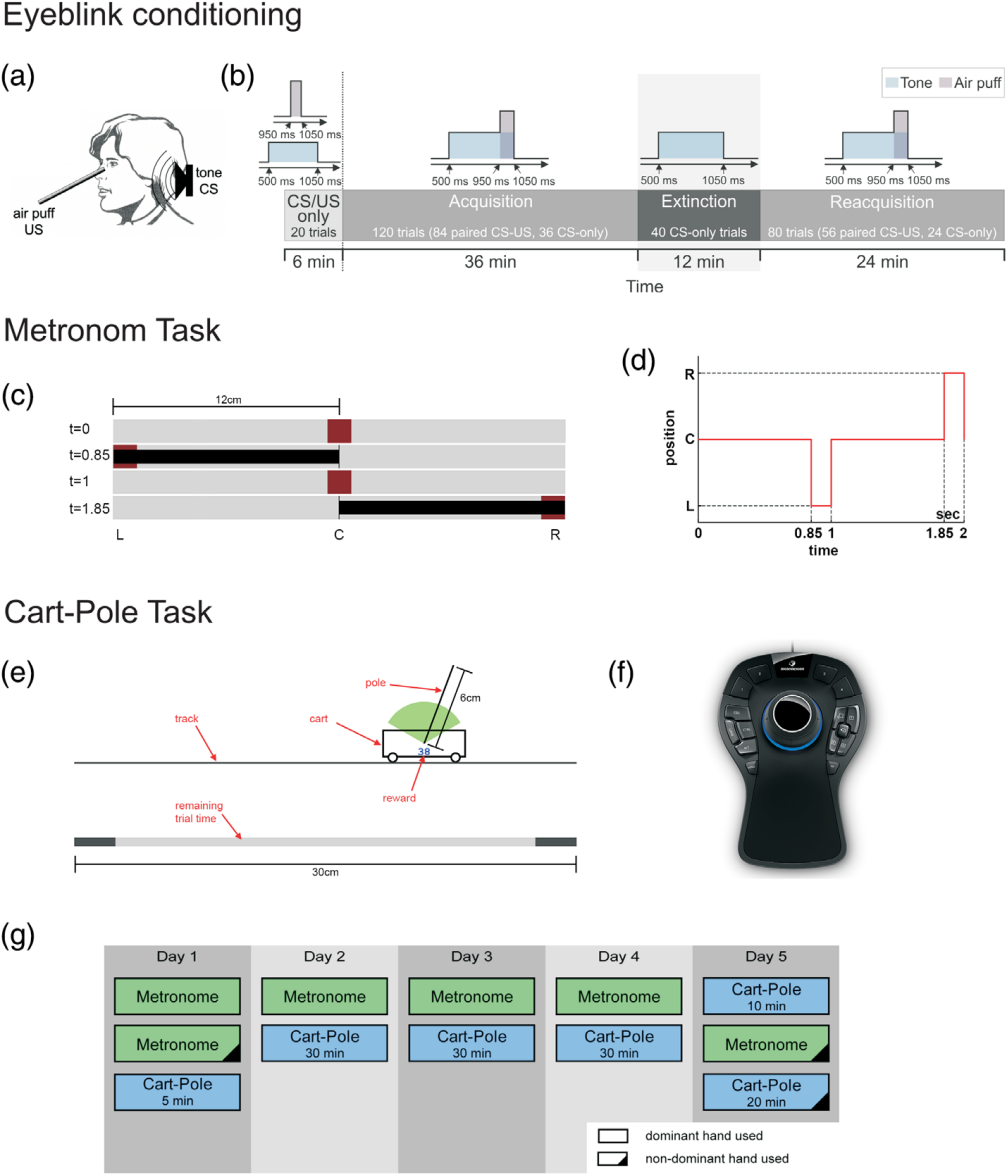
Schematic descriptions and experimental paradigms of the different motor tasks. Eyeblink conditioning: (a) Schematic illustration, (b) experimental paradigm (reproduced from Ernst et al., 2016). Metronome task: (c) Snapshot of the task, and (d) timing schedule. Cart‐pole balancing task: (e) Snapshot of the task, (f) haptic input device for the metronome and cart‐pole tasks. (g) Experimental schedule for the metronome and cart‐pole tasks over the five consecutive days

A standard short delay eyeblink conditioning task was performed as described in Gerwig et al. (2005, 2003). A neutral tone (duration 550 ms) served as conditioned stimulus (CS), and an air puff directed to the eye as unconditioned stimulus (US; duration 100 ms). The CS and the US coterminated. After a limited number of paired CS‐US trials healthy participants learn that the CS predicts the occurrence of the US, and the eyelid is closed prior US onset in a precisely timed manner (Gerwig et al., 2005). Conditioned (CR) and unconditioned (UR) eyeblink responses were recorded via surface EMG recordings from the orbicularis oculi muscles. We tested acquisition, extinction and reacquisition of conditioned eyeblink responses. Saving effects correspond to faster reacquisition of conditioned eyeblink responses following extinction as compared to the initial acquisition phase. In the acquisition phase, 84 CS‐US paired trials were presented with 36 additional CS‐only trials interspersed. In the extinction phase, 40 CS‐only trials were shown. In the reacquisition phase, 56 paired CS‐US trials were presented with 24 CS‐only trials interspersed (see Figure 1b). Both the US and the CS were presented on the ipsilateral side of the cerebellar lesion in patients (see Table 1 in Ernst et al., 2016). In paired and CS‐only trials, CRs were identified in the CS‐US interval using in‐house software (see Gerwig et al., 2010 for details). As a measure of CR acquisition, the percentage of conditioned responses (percentage CR incidence) across all acquisition trials was calculated. As a measure of CR extinction, the difference between percentage CR incidence in the last 10 acquisition trials and the last 10 extinction trials was calculated. As a measure of savings, the difference in percentage CR incidence between the first 10 reacquisition and the first 10 acquisition trials was calculated (see Ernst et al., 2016, for details).

**TABLE 1:** Mean behavioural measures for the eyeblink conditioning, metronome and cart‐pole balancing tasks split by measure and group (cerebellar and healthy control participants)

		Cerebellar participants	Healthy controls
**Eyeblink conditioning**			
(% CR incidences)	Acquisition	20.16 (2.50)	38.77 (2.77)
	Extinction	13.89 (3.18)	28.33 (4.42)
	Savings	8.89 (1.94)	22.78 (2.82)
**Metronome task**	M‐SE	56.51 (11.60)	30.71 (5.28)
**Cart‐pole balancing task**	CP‐AT	1.81 (0.22)	1.24 (0.14)

*Note: SE* of the mean within parentheses.

Abbreviations: CP‐AT, cart‐pole action timing; CR, conditioned response; M‐SE, metronome synchronisation error.

#### 2.2.2 Motor action timing in the metronome and the cart‐pole balancing task

We examined the influence of focal lesions of the cerebellum on rhythmic action timing in the metronome task and the capability of predictive timing in a dynamic object manipulation task (the cart‐pole task).

In the metronome task, participants had to perform actions in synchrony with a predictable visual stimulus to test their elementary predictive action timing capabilities. Specifically, a red dot jumped between three positions on the screen: centre, left and right (Figure 1c). Participants controlled the extent of a black bar measured from the center using a haptic input device (3Dconnexion SpaceMouse® Pro, Figure 1d). The goal of the metronome task is to keep the end of a black bar always within the target red dot. Performance is measured by the average synchronisation error (SE), The SE was calculated by comparing the time of the movement onset with the time of the target's position change in the pattern via subtraction, yielding 150 signed onset errors per block and subject (5 repetitions × 30 movements).

In the cart‐pole balancing task, participants had to learn to control a virtual simulation of the cart‐pole system (Figure 1e). The goal was to keep the pole upright. Participants accelerated the cart by applying virtual forces using a haptic input device. A trial was considered successful if balance was maintained for 30 s. The difficulty of the motor task can be changed by adjusting the gravitational constant in the virtual task. In order to reduce the initial difficulty, the task started with the gravitational constant set to g_init_ = 1.0 m/s^2^. After every successful trial, the gravity was increased by 0.1 m/s^2^ until the maximum of g_max_ = 3.0 m/s^2^ was reached. Thus, due to this performance‐dependent increase, every participant was exposed to an individual gravity profile over the course of the experiment. Overall, the experimental protocol involved five consecutive days. On the first day, we let participants get a first impression (5 min) of the cart‐pole balancing task. The subsequent 3 days (day 2–4) were used for the main cart‐pole balancing training (30 min per session). After the cart‐pole balancing training, the last day (day 5) was devoted to examining the transfer to the non‐dominant hand. Here, participants performed the cart‐pole balancing task again using their dominant hand for 10 min and then switched to their non‐dominant hand to perform the task for another 20 min (Ludolph et al., n.d. bioRxiv).

We identified the measure of action timing (AT) as a crucial prerequisite to master the motor skill acquisition of the cart‐pole task. In order to obtain this measure, we examined the virtual forces that each subject applied as a function of the system state, specifically as a function of the pole angle and the sign of the pole velocity. Calculating this measure involves the definition of events in state space (pole angle and pole velocity) and analysing the applied force and their timing relative to those events (event‐triggered averaging). By averaging these time estimates across all events, we obtained a measure of general action timing performance in the cart‐pole balancing task. In addition, the combination of action timing measures for both hands—AT combined hands—can be interpreted as general action timing capability regardless of the hand used. The full computational details of this method can be found in Ludolph et al., n.d. (bioRxiv).

### 2.3 Structural MRI data acquisition and lesion‐symptom mapping

T1‐weighted MPRAGE images were obtained using a 3 T MRI scanner (Magnetom Skyra, Siemens, Erlangen, Germany) with a standard 32‐channel head‐coil. A number of 192 sagittal slices were acquired with the following parameters: TR = 2,500 ms, TE = 4.38 ms, acquisition matrix 256 × 256, slice thickness = 1 mm; voxel size = 1 mm^3^. In addition, 3D‐FLAIR images were acquired (256 transversal slices, TR = 5,000 ms, TE = 395 ms, acquisition matrix = 256 × 256, slice thickness = 1 mm; voxel size = 1.0 mm^3^). T1 images were used to manually trace cerebellar lesions (surgical cavity) on all axial, sagittal and coronal slices. The resulting masks were then adjusted based on lesion extent in FLAIR images and saved as regions of interest (ROI) using MRIcron (http://www.mccauslandcenter.sc.edu/mricro/mricron) (see Ernst et al., 2016 for details). We computed voxel‐based lesion‐symptom mapping (VBLSM) maps which requires lesions to be in standard space. We therefore applied the deformation parameters calculated during the normalisation of the cerebellum to the lesion mask using a voxel size of 1 mm^3^. For the analysis per se, we implemented a multivariate approach based on support vector regression (SVR), which has the advantage that it identifies only those voxels whose mutual information is associated with the behavioural impairment (Zhang, Kimberg, Coslett, Schwartz, & Wang, 2014). The relationship between the symptom and the whole lesion map was modelled using a nonlinear function, which accounts for intervoxel correlations. To control for the effect of total lesion volume, the ‘direct total lesion volume control’ approach was used, in which each lesion map was normalised to have a unit norm. Statistical analysis was performed using permutation tests (1,000 permutations), and the resulting statistical maps were thresholded at FDR q = 0.05.

### 2.4 DTI data acquisition and analysis

The DTI data were acquired using the following parameters: 60 diffusion directions, b = 0, 1,000 s/mm^2^, TR = 13,300 ms, TE = 92 ms, voxel size = 1.7 mm^3^. Preprocessing of DTI images was performed in FSL and MRtrix. Diffusion images were first brain‐extracted. Then, denoising of diffusion images and noise map estimation were performed. This was followed by eddy current correction, which corrected for movement between acquisitions. Outlying slices (average intensity <4 *SD* lower than expected intensity) were removed and replaced with predictions made by the Gaussian Process. The quality of our data sets was inspected using a series of quality control metrics derived from FSL's eddy_quad function.

For ROI‐based analysis of cerebellar white matter in native space, we initially aligned all T1 images to the AC‐PC axis, and performed a rigid‐body registration of the non‐diffusion image (b0) of the DTI data set to the aligned T1w scan. Next, the T1w images were used to create a cropped image of the cerebellum, which was subsequently segmented into grey and white matter probability images, using the SUIT toolbox (http://www.icn.ucl.ac.uk/motorcontrol/imaging/suit.htm). In order for the lesion not to affect the normalisation algorithm, we masked the region of the lesion (using a safety margin of ~5 mm) during normalisation. We then inverse‐normalised the SUIT atlas to diffusion space, and applied the resulting deformation parameters to a probabilistic atlas of the cerebellar white matter (van Baarsen et al., 2016). Finally, we inversed the co‐registration matrix computed above and applied this transformation to the probabilistic atlas. Thus, in the end, the diffusion image of each individual had an associated probabilistic atlas of cerebellar white matter with which ROIs could be extracted.

Individual FA maps were thresholded at >0.4, as lower values were mostly caused by minute imperfections in registration, consequently spilling the mask into grey matter. In addition, thresholding the FA images improved SNR (Smith et al., 2006), and effectively prevented the inclusion of confounding factors such as previous haemorrhage, which was present in three subjects with vascular tumours (see Figure S1).

**FIGURE 2:**
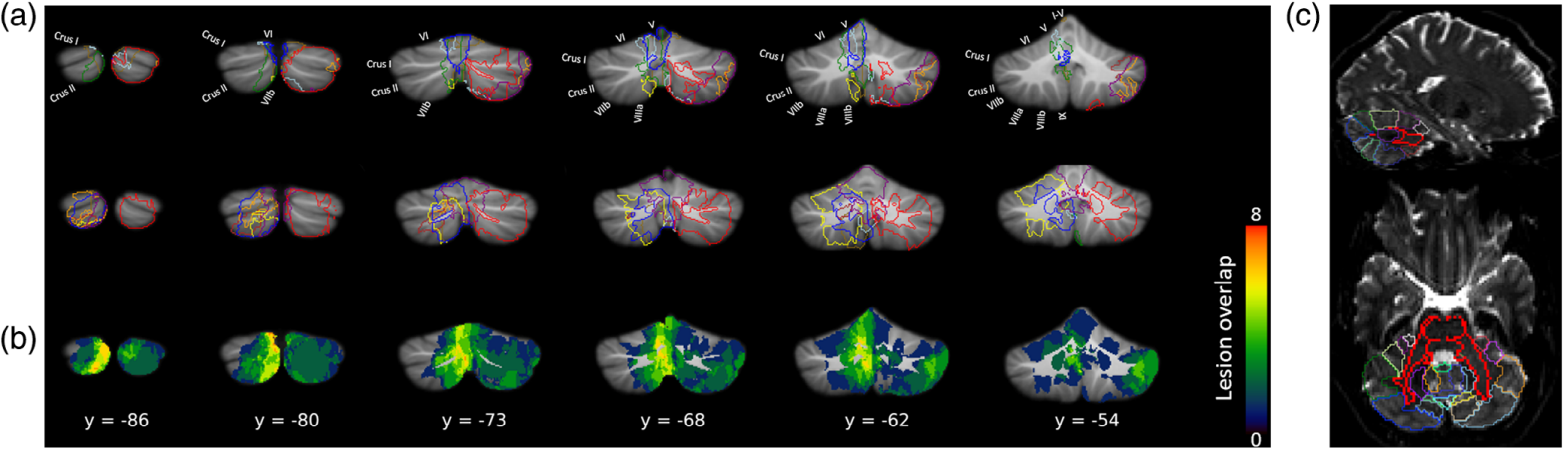
Cerebellar lesion extent displayed on a normalised cerebellum. (a) Outline of lesion for the first nine (top row) and last nine (middle row) cerebellar participants. (b) Lesion overlap. Colourbar indicates the number of patients with lesions on those voxels. (c) SUIT template (Diedrichsen, 2006) overlaid on top of one representative participant's B0 image in diffusion space, showing accurate registration of the lobules (coloured outlines) and cerebellar peduncles (bold red outlines). Note: Lesions are displayed in the original hemisphere in which they occurred. For the analysis of voxel‐based lesion symptom mapping, the left‐hemisphere lesions were flipped horizontally to the right hemisphere

Visual inspection of our masks showed to include predominantly white matter (see Figure 2c). For each measure of the eyeblink conditioning, metronome and cart‐pole balancing tasks, multiple linear regressions were computed using behavioural measures as the dependent variable and mean FA, group and cerebellar peduncle region (inferior, middle and superior) as predictors. Age, sex and motion parameters derived from the eddy current correction were used as covariates in all models.

#### 2.4.1 Tract‐based spatial statistics

For the analysis of DTI data, we fitted a diffusion tensor model at each voxel on the preprocessed data. These were used to produce maps of the different diffusivity metrics: fractional anisotropy (FA), mean diffusivity (MD), axial diffusivity (AD), radial diffusivity (RD) and mode of diffusion (MO). FA quantifies the degree of anisotropy; it can vary between 0 (isotropic diffusion) to 1 (anisotropic diffusion), and it is believed to be a global measure of white matter integrity. MD represents the average diffusion over all directions ([λ_1_ + λ_2_ + λ_3_]/3) and indicates water motility. AD quantifies the amount of diffusion along the principal axis of diffusion (λ_1_), and it can be considered a measure of axonal injury. RD quantifies the amount of diffusion perpendicular to the direction of primary diffusion ([λ_2_ + λ_3_]/2), and it is a measure of demyelination. Finally, MO indicates the shape of the ellipsoid, which can vary between planar (MO = −1) and tubular (MO = 1).

For voxel‐wise analysis of the DTI data, we modified the standard tract‐based spatial statistics (TBSS) pipeline to make it more suitable for working with lesion data. First, we eroded the FA images slightly and removed outliers from the diffusion tensor fitting. Then, we altered the default masks by combining the automatically generated mask with the manually traced mask in DTI space. These masks were used in the subsequent normalisation of FA images to MNI space, in order to mask lesioned voxels and facilitate registration. After the mean FA skeleton was generated, it was thresholded to 0.3, producing a skeletonised version of the image projected onto the white matter skeleton. To exclude the lesioned voxels from the TBSS analysis we adopted the following steps: for each subject, we registered the B0 image to the T1 image and inverted this registration matrix. We then applied the resulting anatomical‐to‐diffusion transformation to our lesion masks. Subsequently, we brought all lesion masks to FMRIB58 space, and projected them onto the TBSS skeleton. After binarising these masks, we passed them to FSL's function ‘setup_masks’, in order to exclude lesioned voxels that do not contain useful data. This function creates modified design matrices and contrasts as well as voxelwise regressors suitable for subsequent analysis. Statistical analysis was performed using FSL's randomise tool (with the threshold‐free cluster enhancement [TFCE] method) using the updated design matrix and contrasts from the “setup_masks” command. The design matrices consisted of behavioural scores, as well as covariates of group, age and estimated motion parameters from eddy current correction. Multiple comparisons were corrected for by controlling the family‐wise error rate (FWE‐corrected *p* < .05). Given that our sample was relatively small, we added a variance smoothing option (5 mm) to the randomise call in order to improve the estimation of the variance in the final statistical images. TBSS was also applied to the other DTI metrics (MD, RD, AD and MO). Having completed the TBSS analysis on the FA data, we ran the “tbss_non_FA” script provided in FSL, which applies the nonlinear registration to the new data, and projects them into the mean FA skeleton computed above.

## 3 RESULTS

### 3.1 Behavioural analysis

Table 1 summarises behavioural data for the eyeblink conditioning, the metronome and the cart‐pole balancing tasks. A mixed model linear regression analysis was conducted in order to compare overall performance between healthy control and cerebellar participants in the three measures representing acquisition, extinction and savings effects in the eyeblink conditioning experiment. We used behavioural scores as the dependent variable and group and measure as the predictors of interest while controlling for age and sex. Participant was treated as a random effect. There was a significant effect of group, F(1,32) = 7.27, *p* = .011, which indicated worse performance in general for cerebellar participants than controls (see Table 1). The group × task interaction was non‐significant, F(1,284) = 0.83, *p* = .439, suggesting similar impairments across all three measures.

Similarly, for the metronome and car‐pole balancing tasks, we only found a significant main effect of group, F(1,29.21) = 4.20, *p* < .05, which, once again, indicated worse performance for cerebellar participants relative to controls (see Table 1). No other effect reached statistical significance (*F*s < 3.92, *p*s > .05).

We also added the covariates of age at diagnosis and handedness, but the significance of the results did not change and their inclusion had no impact on the other effects of interest (*p*s > .26).

### 3.2 VBLSM

For VBLSM, we conducted a multivariate analysis using support vector regression (SVR) (Zhang et al., 2014). Of note, this analysis goes beyond the subtraction method which has been reported in Ernst et al. (2016) by increasing sensitivity (see Methods). Figure 2a,b show the normalised lesion maps across cerebellar participants, which differed in terms of both extension and location. One limitation of VBLSM is that patient samples are normally small and lesions vary considerably both in location and extent. Thus, to increase lesion overlap, and thus statistical power, lesioned voxels on the left hemisphere were flipped along the midline (Ernst et al., 2016; Meyer et al., 2016), such that all lesioned voxels were represented in the right hemisphere (note that in Figure 2, all lesions are shown in the cerebellar hemisphere in which they actually occurred).

**FIGURE 3:**
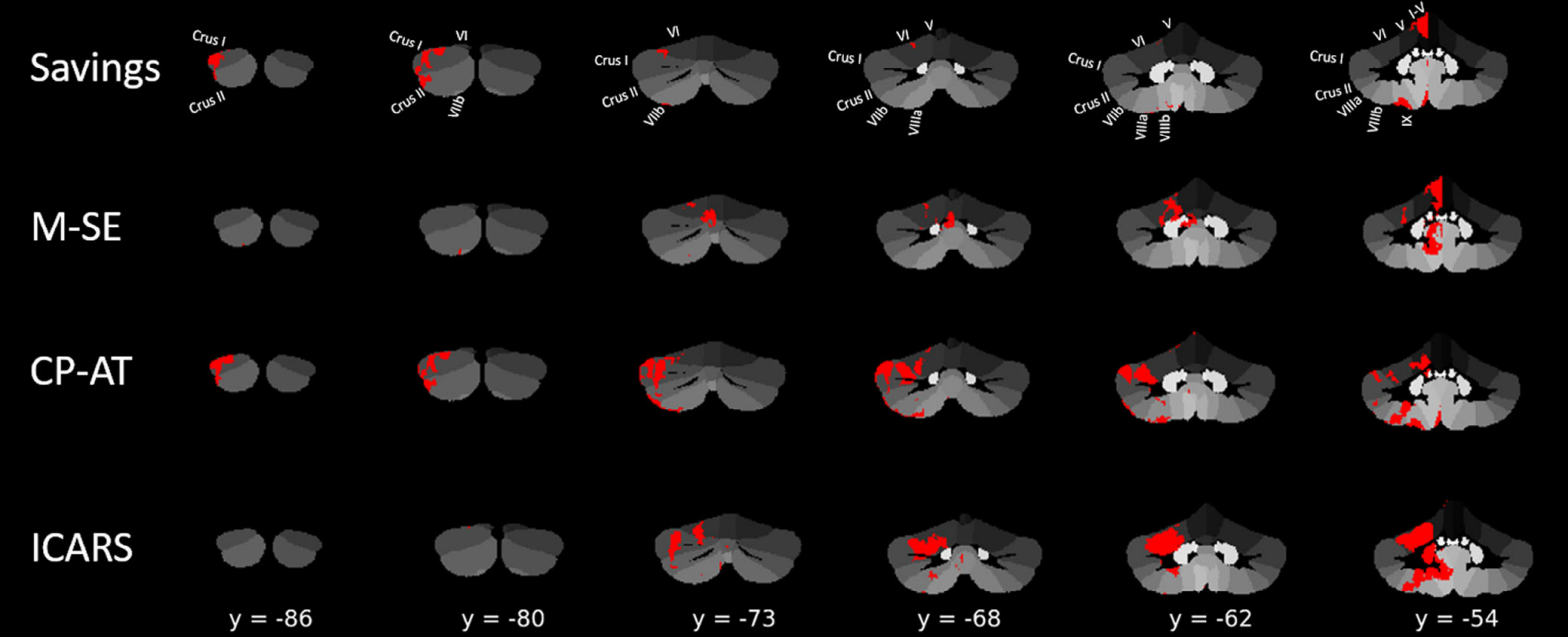
Results of the multivariate voxel‐based lesion‐symptom mapping (VBLSM). For the eyeblink conditioning task, only the Savings parameter showed significant correlations. CP‐AT, Cart‐Pole Action timing; M‐SE, Metronome Synchronisation error; ICARS, International Cooperative Ataxia Rating Scale (Trouillas et al., 1997)

In the eyeblink conditioning task, permutation tests revealed significant correlations only regarding the savings measure (*p* < .05, FWE‐corrected). Saving effects were significantly reduced in cerebellar participants with lesions in the anterior cerebellum (lobules I‐IV, with small extensions into lobules V and VI), the posterior cerebellum (lobules VIIIa, VIIIa and IX) as well as posterolateral areas (Crus I and II) (see Figure 3).

For the metronome task, reduced performance (larger synchronisation error) was associated with lesions of the cerebellar midline, particularly in cerebellar lobules I‐IV, VI and IX. In contrast, for the cart‐pole balancing task, we found the strongest associations between decreased action timing performance and several subregions in the cerebellar hemispheres of the cerebellum, particularly in Crus I‐II, and lobules VIIIa and VIIIb.

Finally, the results for the clinical ataxia (ICARS) scores were, with few exceptions, comparable to those obtained for the learning measure of the cart‐pole balancing task (both in extent and location; see Table S1). They involved the known motor areas of the cerebellum (lobules I‐IV, V, VI, VIII) and extended into Crus I and II and also the dentate nucleus.

### 3.3 ROI analysis of cerebellar white matter

Masks of the inferior, middle and superior peduncles were generated in DTI space for each participant as described in the Methods section. For each measure of the eyeblink conditioning, metronome and cart‐pole balancing tasks, separate multiple linear regression analysis were conducted for FA, MD, AD, RD and MO maps.

**FIGURE 4:**
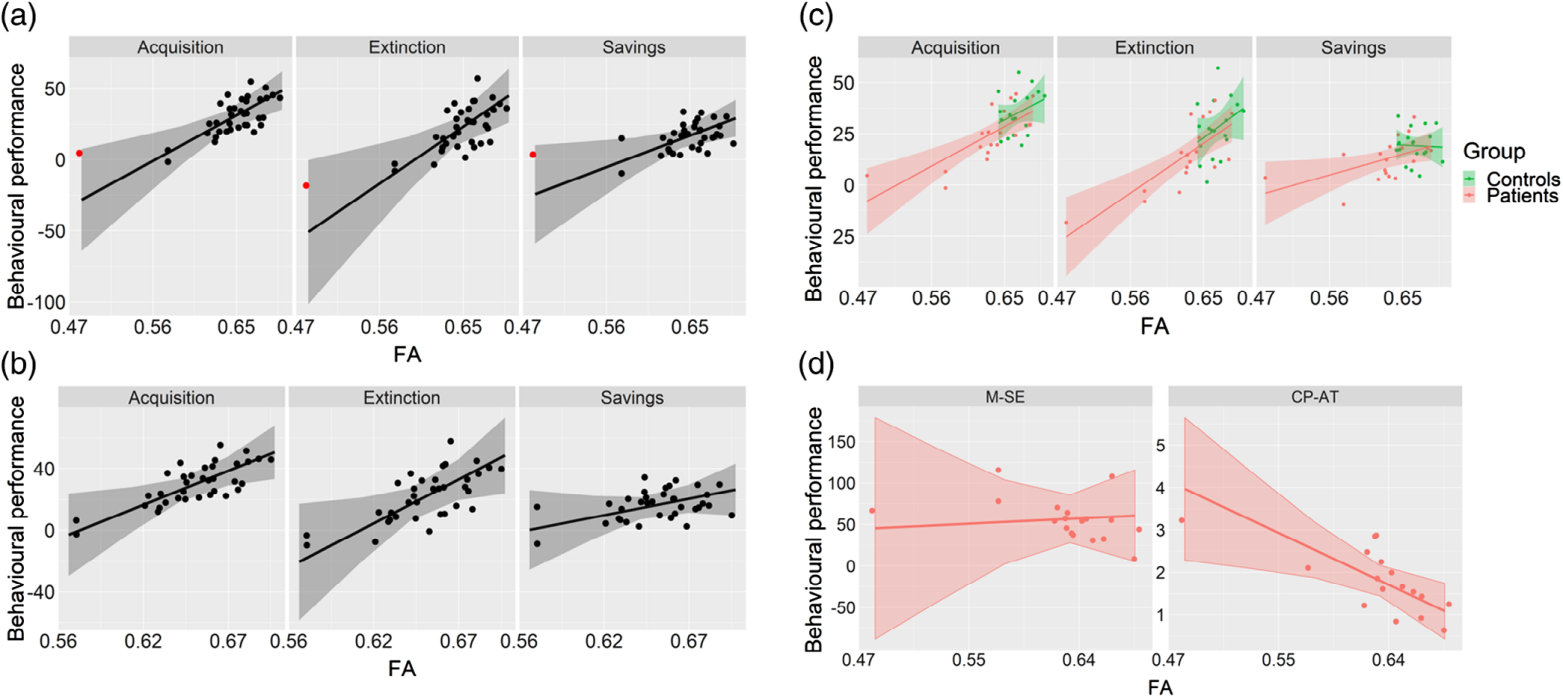
(a–c) Correlations between FA and behavioural performance within the superior peduncle for the eyeblink conditioning task. (a) Identification of an apparent “outlier” (red dot). (b) Removal of the “outlier” did not substantially change the resulting slopes and associated standard errors. (c) Same plot as (a) but colour‐coded to distinguish between cerebellar and control participants' performance. (d) Correlation between FA and behavioural performance for the metronome (M‐SE) and cart‐pole balancing tasks (CP‐AT)

Because the superior cerebellar peduncle (superior peduncle, sPeduncle) is the main efferent fibre tract of the cerebellum and a core component of the cortico‐ponto‐cerebello‐thalamo‐cortical loop (Ramnani, 2006), we focused the regression analysis on this cerebellar peduncle. Regarding the eyeblink conditioning task, there was no interaction between group (cerebellar vs. control participants) and superior peduncle for any of the three learning measures, so a new model was fit including only main effects. For all three models (i.e., considering acquisition, extinction and saving effects), we found a significant main effect in the superior peduncle (all Fs > 5.86, *p*s < .03). This indicated that FA values in this area showed a positive relationship with behaviour, in the sense that the greater the FA, the better the performance predicted by the model, and this effect was not specific to any particular group (see Figure 4a,c). Interestingly, neither the middle peduncle nor the inferior peduncle were associated with any of the behavioural measures (all Fs < 4.08, *p*s > .05).

For the cart‐pole balancing task, there was also a significant relation between FA values in the superior peduncle and the CP‐AT parameter, F(1,9) = 8.86, *p* = .016, indicating that better task‐related action timing performance (as indicated by smaller values) was associated with an increase in mean FA (Figure 4d). For the model with the metronome task (M‐SE), there were no significant effects (Fs < 1.32, *p*s > .27).

One may argue that the effects described above were strongly influenced by a single case (red dot in Figure 4a) that could have influenced the regression slopes. However, this case was not an influential case in the statistical sense, since the Cook's distance was well below 1 in all cases (< 0.3). In fact, the regression slopes changed little with the removal of this particular participant (see Figure 4b).

There was no significant relationship between the behavioural measures and the other DTI metrics (MD, AD, RD and MO) within the superior peduncle.

### 3.4 TBSS analysis

**FIGURE 5:**
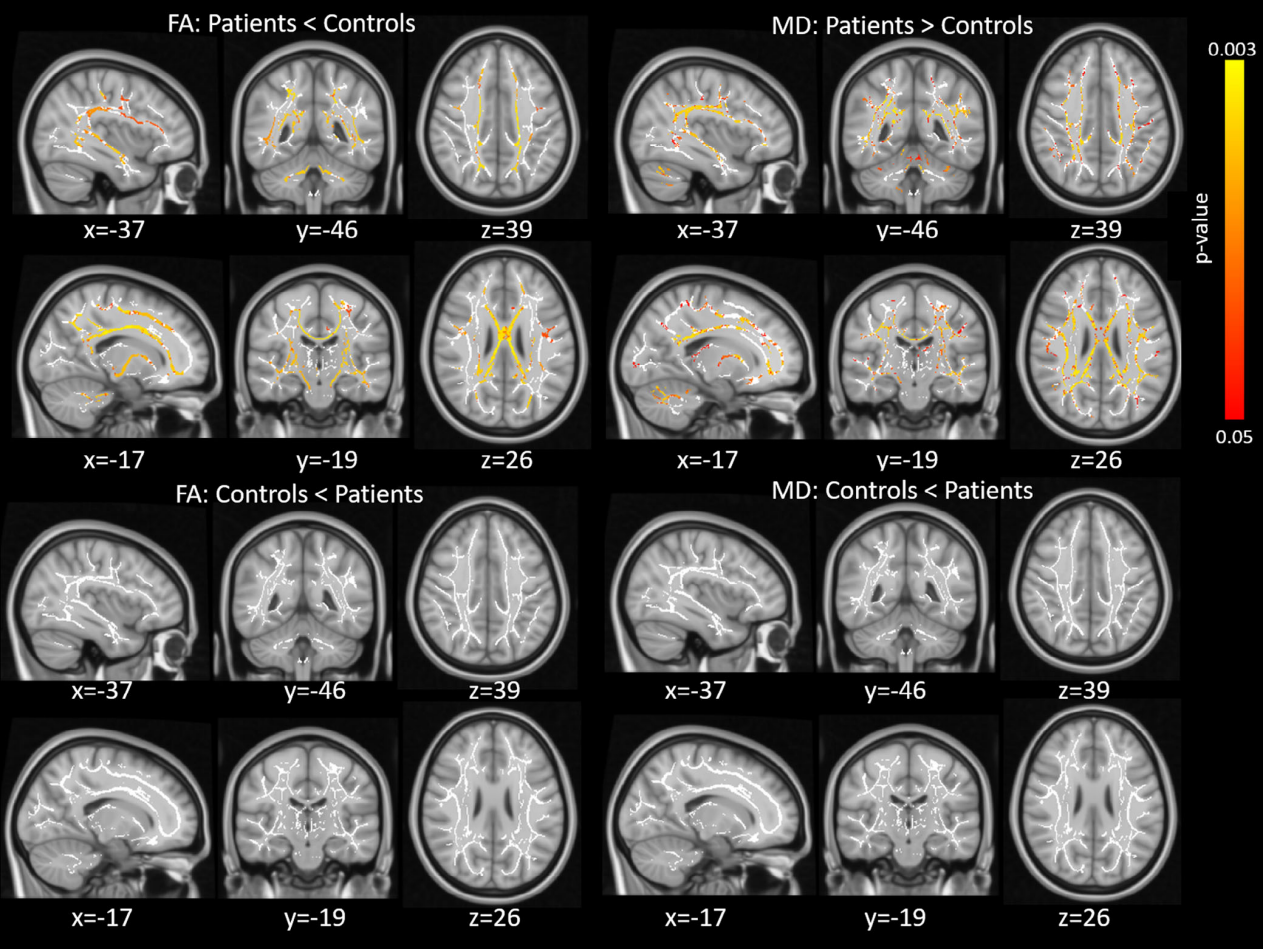
Tract‐based spatial statistics (TBSS) results for the group comparison. Top two rows show the results for the contrast cerebellar < control participants for the FA (light) and MD (right) metrics. The bottom two rows show the results for the opposite contrast (cerebellar > control participants). Red‐yellow colour shows which voxels surviving correction for multiple comparisons (FWE‐corrected *p* < .05), which shows the white matter skeleton (threshold 0.3–0.7)

Voxelwise statistics were performed using TBSS across all DTI metrics (FA, MD, RD, AD and MO). Appropriate design matrices were created which consisted of the particular contrast of interest (cerebellar vs. control participants) and controlling variables of age, sex and motion estimates derived from eddy current correction. Our initial contrast simply looked for voxels that showed greater FA values in controls than in cerebellar participants. A vast network of tracts showed reduced FA for cerebellar relative to control participants (see Figure 5, left). These tracts included, not only part of the cerebellum (where the lesions were restricted to), but also distant cortical tracts. Furthermore, both MD and RD also showed strong effects between cerebellar and control participants, with the direction of the contrast reversed (i.e., cerebellar participants showed greater values of MD/RD relative to controls; see Figure 5, right). MD measures overall diffusivity in the tissue, with lower values commonly associated with greater structural organisation, whereas higher RD values are believed to indicate myelin injury (Song et al., 2003; Song et al., 2005). The statistical maps showing lower FA for cerebellar versus control participants were strikingly similar to the maps showing higher MD as well as higher RD (see also Table S2).

The opposite contrast of greater FA (or lower MD/RD) for cerebellar relative to control participants revealed no significant voxel.

**FIGURE 6:**
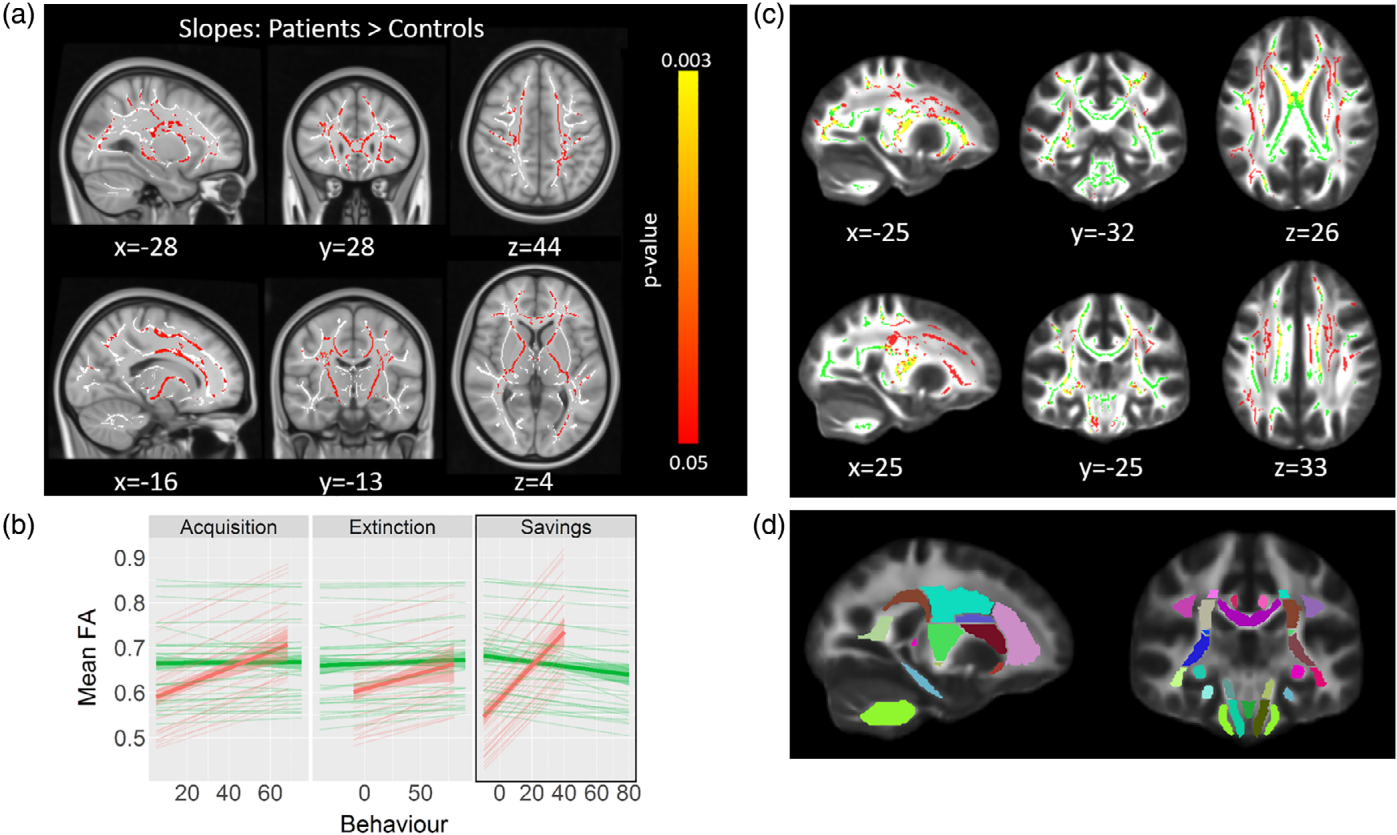
Tract‐based spatial statistics (TBSS) results comparing group slopes with fractional anisotropy (FA) values. (a) Results for the contrast testing the cerebellar > control participants' slopes for the Savings measure of the eyeblink conditioning task. Red‐yellow colour shows voxels surviving correction for multiple comparisons (FWE‐corrected *p* < .05), white shows the white matter skeleton (threshold 0.3–0.7). (b) Voxel values in (a) were extracted and FA values plotted against behavioural scores for each of the extinction learning measures. Thick lines show the overall direction of the effect (i.e., across all significant voxels). Thin lines show the regression slopes for each individual ROI (with >30 significant voxels; see also Table S3). (c) Overlap between the contrast map shown in (a) (red) and the cerebellar < control participants contrast from Figure 4 (green). Yellow shows voxels that were common in both contrasts. (d) Overlay showing the 48 white‐matter tracts from ICBM‐DTI‐81 atlas (Mori et al., 2008), which were used to create the figure in (b)

Next, we performed linear analyses, in which we tested which voxels showed a linear relationship between the dependent variable (DTI metric) and the behavioural measures. Figure 6 shows the results of these analyses.

We examined whether the slopes indicating the behavioural relevance of FA values differed significantly between cerebellar participants and controls in these voxels. Regarding the eyeblink conditioning task, the Savings measure revealed strong effects in three of the DTI metrics: FA, AD and MO. Specifically, the FA and AD statistical maps shared many of the same tracts (see Figure 6), including the body and genus of the corpus callosum, corona radiata, internal and external capsule, longitudinal fasciculus, fornix, thalamic radiation, sagittal stratum and cingulate gyrus. Overall, this effect suggests that cerebellar participants showed a more positive relationship between behaviour and FA than controls, whose slopes did not differ from zero (see Figure 6b). Although the voxel extent tended to vary considerable between metrics, the pattern of results was similar (see Table S2). The opposite contrast inquiring which voxels showed a larger slope for controls than for cerebellar participants revealed no significant voxel. Even though there was no difference between groups in brain‐behaviour relationship with either the Acquisition or Extinction parameters, the direction of the regression slopes were highly similar among the three behavioural parameters (see Figure 6b).

**FIGURE 7:**
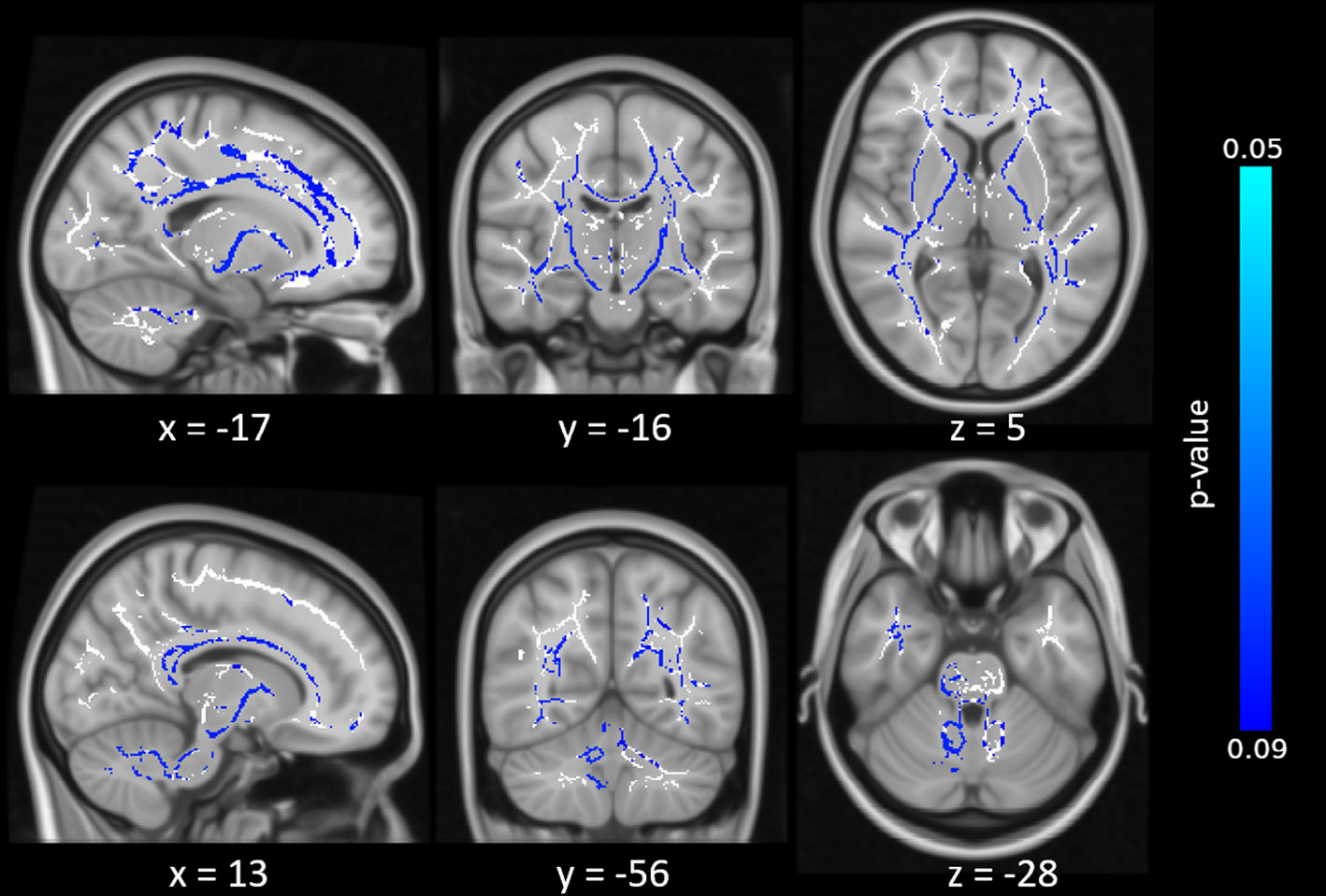
Tract‐based spatial statistics (TBSS) results for the cerebellar participant group only, testing which voxels show a correlation with the M‐SE. Blue colour indicate voxels that were short of significance (*p* < .09) after correcting for multiple comparisons, white shows the white matter skeleton (threshold 0.3–0.7)

For the metronome and cart‐pole balancing tasks, we did not find any relationship between behavioural and voxel‐wise FA or any other DTI metric. However, this was likely due to the small sample, since we could only include the 18 cerebellar participants (there were no MRI scans available for the controls in these two tasks). In fact, looking for voxels showing marginal effects (0.05–0.09 significance level range) we did observe an extensive network of tracts that were just short of significance for the metronome task M‐SE parameter (see Figure 7 and Table S2).

## 4 DISCUSSION

In this study, we analysed diffusion weighted imaging data from cerebellar patients with chronic surgical lesions of the cerebellum. Voxel‐wise analysis of white matter integrity showed widespread white matter changes in young adults after tumour resection in childhood. White matter changes of the superior cerebellar peduncle, the main outflow pathway of the cerebellum, but also many cerebral white matter pathways, were related with action timing and motor learning deficits. These findings confirm and extend previous findings of long‐term sequelae of posterior fossa surgery and will be discussed in detail below.

#### 4.1 Surgery for benign cerebellar tumours results in widespread infra‐ and supratentorial white matter changes

A vast network of white matter tracts showed reduced fractional anisotropy (FA) in patients compared with controls beyond the cerebellar white matter. This finding indicates that cerebellar lesions due to resection for benign cerebellar tumours at an early age cause extensive network damage.

Our findings are in good accordance with previous DTI studies showing significant FA changes at several supratentorial tracts, in particular the cerebello‐thalamo‐cortical tract. For example, Morris et al. (2009) found that patients who developed posterior fossa syndrome following childhood cerebellar tumour resection showed reduced FA not only in the cerebellar peduncles, but also in the fornix and in a cluster near the posterior corona radiata. Rueckriegel et al. (2010) tested paediatric survivors of cerebellar tumours (with and without adjuvant therapy) and also found significant changes in FA values beyond the cerebellum, particularly in several frontal lobe tracts, as well as in the callosal body. Similarly, Soelva et al. (2013) used tractography to investigate differences between patients (also with and without adjuvant therapy) and controls within the fronto‐cerebellar course. They defined an ROI comprising the white matter of the vascular territory of the frontopolar artery, the frontal anteromedial artery and the anterior limb of the internal capsule. The results showed a clear reduction in FA for patients relative to controls. We observed reduced FA in all the tracts mentioned in the lesion studies above (see Figure 5 and Table S2). These findings agree with, and further support, virus tracing studies in monkeys (Bostan, Dum, & Strick, 2013) and DTI studies in healthy controls (Piervincenzi et al., 2017; Tavor, Botvinik‐Nezer, Bernstein‐Eliav, Tsarfaty, & Assaf, 2020; Wang, Casadio, Weber, Mussa‐Ivaldi, & Parrish, 2014) providing evidence that the cerebellum has efferent connections to widespread cortical areas in the frontal lobe, but also the parietal and occipital lobe.

The lesion studies described above showed more modest white‐matter deficits for cerebellar participants than controls, although this could be partly attributed to the different methodologies used. Specifically, we employed a DTI analysis strategy which was optimised to deal with lesion data, whereas most DTI studies of cerebellar participants used default pipelines that might be less sensitive to detect differences between groups. In addition, most of the aforementioned studies used an ROI‐based approach which limits direct comparison with the present whole‐brain and voxel‐level results.

Patients showed reduced FA values, as well as increased MD, relative to controls. The opposite directionality for these two metrics is in accord with what would be expected from both animal and human models. The progressive destruction of the cytoskeleton that normally hinders water diffusion results in the degeneration of the part of the axon distal to the site of the lesion (Wallerian degeneration). The disintegration of these barriers, concomitant breakdown of myelin sheaths and phagocytosis by microglial cells allow water to diffuse in all directions (isotropic diffusion), reducing axonal directionality, which, in DTI, is detected as a decrease in FA (and increase in MD).

FA and MD are summary metrics which provide a general and useful measure of maturation and integrity of white‐matter, but they do not allow to discriminate between axonal or myelin pathology. Specifically, MD represents the average of parallel (λ1, AD) and perpendicular (λ2, λ3, RD) diffusivity to the principal axis of the fibres. Animal studies show negative correlations between RD and myelination, whereas decreases in AD are often concomitant with axonal damage (Song et al., 2005, Song et al., 2003, 2002). In our study, the contrast between patients and controls revealed changes not only in FA/MD but also in RD, suggesting that cerebellar lesions relate to myelin disintegration across many of the extracerebellar tracts. Gradual loss of white‐matter integrity, particularly demyelination, in supratentorial tracts may be related to the process of anterograde transsynaptic degeneration (Dinkin, 2017; Triarhou, Norton, & Ghetti, 1987). Here, following interruption of the anatomical pathway due to axonal damage of a cell, neuronal degeneration may spread to distant neurons that serve the same function as the injured neuron. Aberrant electrical and/or chemical activity arising from the site of injury can cause damage to distal brain areas functionally connected to the damaged brain region (a phenomenon known as diaschisis). This relates to the principle that, being integrated into a functional network, neurons need to receive trophic signals from other functionally‐related neurons in order to be sustained. If inflammation occurs as the result of this process, it may trigger abnormal activation of astrocytes, which further degenerate mature oligodendrocytes—the type of glia cells responsible for supporting myelination (Nave & Werner, 2014; Sofroniew, 2005). Indeed, studies involving patients with hippocampal sclerosis, for example, have found that atrophy in distant areas of the primary injury site was increased in several regions of the Papez circuit after surgery, which likely indicated interrupted efferent axonal activity due to the surgical intervention (Kim, Tien, Felsberg, Osumi, & Lee, 1995; Kodama et al., 2003).

In sum, the present data confirm that the cerebellum has extended connections with many cerebral areas involved in motor and cognitive functions (Strick, Dum, & Fiez, 2009), and the observed white matter changes likely contribute to the motor and cognitive dysfunction of posterior fossa tumour survivors (Rueckriegel et al., 2015). Relationships between white matter changes and action timing and motor learning deficits are discussed in more detail below.

#### 4.2 Correlation of cerebellar grey and white matter integrity with action timing and motor learning

Our group and others have shown that lesions primarily of cerebellar lobule VI and, to some extent, of Crus I and Crus II result in reduced acquisition of conditioned eyeblink responses in humans, which is in very good accordance with the animal literature (Ernst et al., 2017; Gerwig et al., 2003). In the present study, these findings were extended to correlations between eyeblink conditioning parameters and white matter integrity in the superior cerebellar peduncle and also cerebral white matter tracts. For both participants with chronic surgical lesions of the cerebellum and healthy controls a significant positive correlation was found between acquisition, extinction and savings of conditioned eyeblink responses and white matter integrity of the superior cerebellar peduncle, the main outflow pathway of the cerebellum. Although these correlations appeared to be more prominent in cerebellar participants than controls, there were no significant differences between the groups.

Correlations between white matter integrity of the cerebellar peduncles and motor learning in healthy human participants have also been reported by others. Della‐Maggiore and colleagues found that FA in the superior cerebellar peduncle correlated with learning rate in a visuomotor adaptation task. Jossinger, Mawase, Ben‐Shachar, and Shmuelof (2020) found significant correlations between white matter properties in the inferior cerebellar peduncle and locomotor adaptation. We extend these findings to eyeblink conditioning, another motor learning task known to critically depend on the integrity of the cerebellum (Donchin et al., 2012; Gerwig et al., 2003; Rabe et al., 2009).

On the level of the superior peduncle we found correlations with parameters related to the initial acquisition, the unlearning or extinction, and the reacquisition of conditioned eyeblinks. In reacquisition, learning is faster compared with the initial acquisition which is explained by saving effects (Napier, Macrae, & Kehoe, 1992). These three measures are not independent; that is, if no acquisition has occurred in the first place, extinction cannot be assessed. However, even though acquisition effects may be negligible, there may still be effects of savings (i.e., faster reacquisition after extinction has occurred). In other words, saving effects may be less dependent on initial acquisition than extinction effects. Cerebellar and cerebral areas involved in saving effects may be more extended than the regions being most critical for acquisition. In fact, our VBLSM results indicated significant correlations between cerebellar areas and savings, but not with acquisition and extinction. These areas, however, only partly overlapped with the acquisition‐related areas in Ernst et al. (2017). It is also worth pointing out that Ernst et al. used a VBLSM analysis based on a subtraction approach, which is purely descriptive, and a binomial statistical (Liebermeister) test. In contrast, we applied a multivariate VBLSM technique, which provides a statistical means to assess the relationship between brain and behaviour and allows behavioural data to be added as a continuous measure. Being a multivariate approach, this technique has the advantage that it considers spatial correlations among neighbouring lesioned voxels (Zhang et al., 2014). This is important because the lesion symptom map will be more influenced by voxels with a strong lesion‐symptom association, whereas noisy lesion‐symptom associations are effectively suppressed. This results in a much more sensitive way to detect lesion‐symptom relationships, and one possible reason why we were able to identify stronger effects with the savings parameter.

Findings related to the metronome and cart‐pole balancing tasks are also in good accordance with the literature. Both tasks use the same haptic input device (Figure 1f) and the type of performed control actions are very similar. The task‐dependent timing and control requirements, however, are more complex in the cart‐pole balancing task. While the participant has to follow a predetermined rhythm in the metronome task, for performing an adequate action‐timing in the cart‐pole task, the participant has to predict the dynamic behaviour of the cart‐pole system (Ludolph, Plöger, Giese, & Ilg, 2017; Mehta & Schaal, 2002; Wolpert, Ghahramani, & Flanagan, 2001). Healthy human participants are thought to learn and maintain internal forward models of object dynamics for optimising their actions and the corresponding sensory consequences (Blakemore, Goodbody, & Wolpert, 1998; Kawato, 1999; Wolpert & Ghahramani, 2000; Wolpert, Ghahramani, & Jordan, 1995). There is initial experimental evidence that these internal forward models representing the dynamic model of the cart‐pole system are located in more posterior and lateral regions of the cerebellum (e.g., Crus I), which fits well with the VBLSM results we observed for the cart‐pole balancing task (see Figure 3). In contrast, the metronome task requires the timing towards an externally predetermined rhythm, which has been shown to involve the cerebellar hand region in lobules IV–VI (Desmond, Gabrieli, Wagner, Ginier, & Glover, 1997; Stefanescu et al., 2013; Thürling et al., 2015). This difference in functional involvement is also reflected in correlations of white matter integrity in the cerebellar peduncles. We found that the superior cerebellar peduncle (but not the inferior or middle cerebellar peduncles) exhibited a close relationship with the timing performance in the cart‐pole task in that higher FA values correlated with better performance (more explicitly, more predictive action timing) (Figure 4). In contrast, we did not find such a correlation for the metronome task. This might be explained by the more medial locations of performance‐associated regions. While lateral regions of the cerebellum project to the dentate nucleus and efferent projections leave the cerebellum via the superior cerebellar peduncle, more medial regions project to the fastigial nuclei with its major efferent connections via the inferior cerebellar peduncle (Miall, 2013).

In summary, findings at the level of the cerebellum and the cerebellar peduncles are in good accordance with the known functional compartmentalization of the cerebellum.

#### 4.3 Correlations of cerebral white matter integrity with action timing and motor learning

In the eyeblink conditioning task, saving effects showed a significant correlation with the integrity of widespread cerebral white tracts in cerebellar participants but not controls. Thus, saving effects likely depend not only on the cerebellar cortex, but also on cerebral areas. These findings agree with previous imaging and human lesion data. Firstly, in an fMRI study with healthy participants using the same paradigm (Ernst et al., 2017), fMRI activity of the cerebellar cortex did not show significant differences between initial acquisition and, following an extinction phase, reacquisition of conditioned eyeblink responses. It was concluded that saving effects may depend on areas other than the cerebellar cortex, which may be the cerebellar nuclei but also cerebral areas. Secondly, in patients with fragile X syndrome, a disorder which is known to affect the cerebellar cortex, preserved saving effects were shown despite impaired acquisition and extinction of conditioned eyeblinks (Smit et al., 2008). Finally, cerebellar theta burst stimulation did not lead to disordered saving effects in an eyeblink conditioning study in healthy human participants (Monaco, Casellato, Koch, & D'Angelo, 2014).

The present findings provide further evidence that saving effects may be supported by a different and/or more extended cerebello‐cerebral network than acquisition, and possibly also extinction of conditioned eyeblink responses. Correlations in the present study were widespread and included the corpus callosum, corona radiata, internal and external capsule, longitudinal fasciculus, fornix, thalamic radiation, sagittal stratum and cingulate gyrus, white matter tracts known to be involved in motor learning. The present findings show that motor learning deficits in participants with surgical lesions of the cerebellum do not likely result from the cerebellar lesion alone, but that long‐term changes of the cerebral white matter also contribute.

For the metronome and cart‐pole task, we did not find a significant relationship between behavioural parameters and cerebral white matter integrity. However, correlations between the metronome task and an extensive network of tracts were observed at a trend level (see Figure 7 and Table S2). Given that the cart‐pole task was, arguably, the most difficult task, one would have expected that abnormalities in DTI measures would show a stronger correlation with behavioural decrements in this task than any other, but this was not the case. As mentioned above, different areas of the cerebellum are most probably involved in the three tasks. Figure 3 suggests that the maximum lesion overlap existed within the intermediate parts of lobule VI, which is most critically involved in eyeblink conditioning. This could partly explain why the relationship between white matter integrity and behaviour was most prominent for eyeblink conditioning. Furthermore, lack of significance was likely explained by the smaller group size, as there was no MRI data available for the control group of the metronome/cart‐pole task.

Correlations between DTI metrics and savings (and, to a lesser extent, rhythmic action timing) were present in cerebellar but not control participants. We can think of at least two possible explanations for this finding. Firstly, patients tend to exhibit greater variance in FA, as well as behaviour, than controls. Greater heterogeneity in the patient sample is likely, albeit not solely, due to the effects of lesion extent and/or location of the lesion on behavioural performance, leading to a more dispersed brain‐behaviour relationship. Healthy controls, on the other hand, might perform more homogeneous, and show FA values that are less variable as a group. These differences possibly augmented the chances of observing a stronger relationship with cerebellar participants.

Secondly, but not mutually exclusive, the effects seen in cerebellar participants could reflect a compensatory mechanism; a result of an impaired cerebellum, leading to a greater reliance on structures outside the cerebellum. When cortical cerebellar regions relevant for savings are impaired, the brain might primarily rely on relatively preserved brain regions also involved in the task. As a result, extra‐cerebellar regions become more sensitive during reacquisition. This does not occur with the healthy controls, however, likely because cerebellar function is intact in that group, so output from the cerebellum alone could be sufficient to support performance in control participants. Some studies have also found significant correlations between FA and behaviour performance in patients, as well as older subjects, but not control participants (Chen, Chou, Song, & Madden, 2009; Davis et al., 2009; Qiu et al., 2019; Takenobu et al., 2014). In most of these studies, the correlations were observed within tracts/regions that were adjacent to those primarily involved in relevant motor/cognitive functions, and were interpreted as compensation. This suggests that the brain adapts to atrophy by offloading information through indirect pathways, thus maintaining some residual performance. Indeed, correlations between savings and FA in the present study, although widespread, included many of the white matter tracts known to be involved in motor learning (e.g., corpus callosum, corona radiata, internal and external capsule, longitudinal fasciculus, fornix and cingulate gyrus) (Sampaio‐Baptista, Sanders, & Johansen‐Berg, 2018; Wang et al., 2014).

#### 4.4 Limitations of the present study

One obvious limitation of the current study is the relatively small sample size used. Although published articles with samples of less than 20 cerebellar patients are common (Oh et al., 2017; Ojemann et al., 2013; Olivito et al., 2017; Perreault et al., 2014), such small cohorts might raise issues of sensitivity. Note, however, that we had very strict inclusion criteria—we could only include patients who had no extra‐cerebellar injury, received no adjuvant therapy, were German native speakers, and were MRI compatible. For this reason, we were severely limited with the amount of cerebellar participants we could test.

Importantly, however, for the critical TBSS analysis, we implemented a nonparametric approach that allows the application of a smoothed variance *t* test. This method is known to improve the estimation of the variance that feeds into the final statistic image. This ‘variance regularisation’ effectively increases the degrees of freedom (by smoothing the variance image with a specific FWHM), resulting is an increase in power with negligible bias (Nichols & Holmes, 2002).

Nevertheless, we acknowledge that our findings will need to be replicated on a different patient sample.

## 5 CONCLUSION

In summary, our results show that the superior cerebellar peduncle is critical to the optimal conveying of motor learning information from the cerebellum to the cerebrum. In addition, lesions in the cerebellum acquired early in childhood have a widespread impact on white‐matter integrity in many cerebellar‐cortical tracts which contribute to motor learning deficits. The present data confirm that the cerebellum has extended efferent connections with many cerebral areas involved in motor but also cognitive functions, and the observed white matter changes likely contribute to long‐term clinical deficits of posterior fossa tumour survivors.

## Supporting information


**Appendix** **S1**: Supporting Information

## Data Availability

MRI/DTI data cannot be openly shared because it contains patient information.
