# Supplementary material for: Resection of cerebellar tumours causes widespread and functionally relevant white matter impairments
Source: Hum Brain Mapp. 2021 Jan 7;42(6):1641–56. doi: 10.1002/hbm.25317 (PMC7978119; doi:10.1002/hbm.25317)
Supplement: Supplementary file 1 — Appendix S1: Supporting Information [file HBM-42-1641-s001.docx]

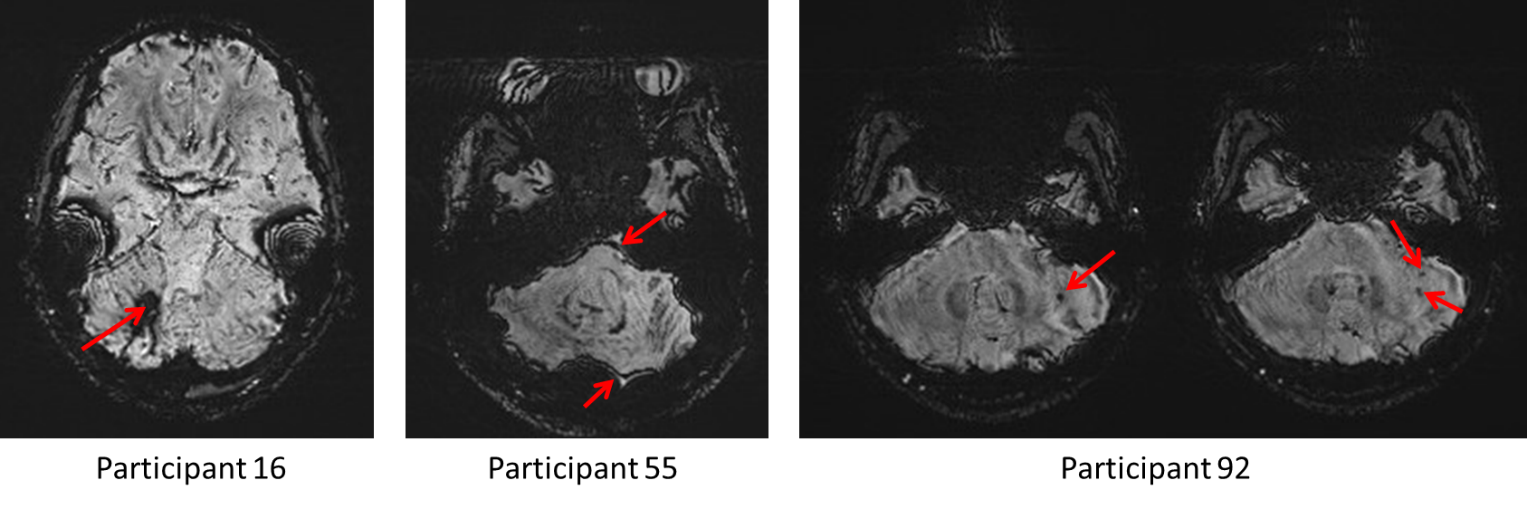


**Supplementary Fig. 1**. Susceptibility weighted imaging (SWI) images of three patients with vascular tumours showing signs of previous bleeding (red arrows). In participant 16, haemorrhage occurred only within the surgical cavity, which was masked during analysis, and, thus, did not affect FA values. In participant 55, there were signs of superficial siderosis, but this had only a negligible effect on the outer rim of the peduncles. In participant 92, haemorrhage occurred outside the cerebellar peduncles and, thus, also did not affect FA.

| **Cerebellar subregion** | **Eyeblink Conditioning** | **Motor learning** | | **ICARS** |
| --- | --- | --- | --- | --- |
|  | **Savings** | **Learning** | **Performance** |  |
| Right_I_IV | 1050 | 486 | 1333 | 507 |
| Right_V | 248 | 1041 | 461 | 1807 |
| Vermis_VI | 121 | 0 | 573 | 0 |
| Right_VI | 164 | 1735 | 1076 | 6275 |
| Right_CrusI | 1095 | 5706 | 19 | 2172 |
| Vermis_CrusII | 4 | 0 | 189 | 19 |
| Right_CrusII | 315 | 1032 | 59 | 65 |
| Vermis_VIIb | 7 | 0 | 147 | 0 |
| Right_VIIb | 22 | 552 | 22 | 147 |
| Vermis_VIIIa | 94 | 0 | 371 | 35 |
| Right_VIIIa | 74 | 1949 | 12 | 1657 |
| Vermis_VIIIb | 15 | 82 | 49 | 1 |
| Right_VIIIb | 674 | 1304 | 2 | 985 |
| Vermis_IX | 212 | 32 | 494 | 10 |
| Right_IX | 371 | 270 | 1451 | 1354 |
| Vermis_X | 104 | 0 | 164 | 23 |
| Right_X | 0 | 80 | 0 | 80 |
| Right_Dentate | 0 | 19 | 240 | 754 |
| Right_Interposed | 0 | 46 | 141 | 2 |
| Right_Fastigial | 0 | 0 | 9 | 0 |
| Right_I_IV | 1050 | 486 | 1333 | 507 |

**Supplementary Table 1.** Number of thresholded voxels that survived correction in the multivariate lesion symptom mapping analysis for the extinction and motor learning experiments, as well as ICARS measure.

| **TOI** | **FA** | **MD** | **RD** |
| --- | --- | --- | --- |
| middle cerebellar peduncle | 1107 | 1498 | 275 |
| pontine crossing tract | 137 | 208 | 0 |
| genu of corpus callosum | 1134 | 1003 | 533 |
| body of corpus callosum | 2930 | 2773 | 1991 |
| splenium of corpus callosum | 2327 | 2111 | 1106 |
| fornix collumn and body | 0 | 51 | 0 |
| corticospinal tract r | 47 | 52 | 0 |
| corticospinal tract l | 57 | 94 | 0 |
| medial lemniscus r | 87 | 39 | 0 |
| medial lemniscus l | 156 | 116 | 0 |
| inferior cerebellar peduncle r | 143 | 137 | 36 |
| inferior cerebellar peduncle l | 145 | 137 | 60 |
| superior cerebellar peduncle r | 214 | 185 | 81 |
| superior cerebellar peduncle l | 182 | 153 | 75 |
| cerebral peduncle r | 290 | 281 | 125 |
| cerebral peduncle l | 383 | 353 | 28 |
| anterior limb of internal capsule r | 484 | 506 | 250 |
| anterior limb of internal capsule l | 486 | 519 | 241 |
| posterior limb of internal capsule r | 837 | 853 | 438 |
| posterior limb of internal capsule l | 831 | 828 | 384 |
| retrolenticular part of internal capsule r | 441 | 479 | 215 |
| retrolenticular part of internal capsule l | 499 | 537 | 269 |
| anterior corona radiata r | 418 | 1279 | 912 |
| anterior corona radiata l | 674 | 1281 | 777 |
| superior corona radiata r | 567 | 1073 | 792 |
| superior corona radiata l | 692 | 1118 | 886 |
| posterior corona radiata r | 340 | 653 | 605 |
| posterior corona radiata l | 320 | 615 | 435 |
| posterior thalamic radiation r | 548 | 626 | 408 |
| posterior thalamic radiation l | 665 | 749 | 536 |
| sagittal stratum r | 347 | 459 | 366 |
| sagittal stratum l | 360 | 392 | 235 |
| external capsule r | 258 | 746 | 435 |
| external capsule l | 558 | 931 | 622 |
| cingulum cingulate gyrus r | 83 | 102 | 198 |
| cingulum cingulate gyrus l | 286 | 293 | 125 |
| cingulum hippocampus r | 1 | 1 | 14 |
| fornix stria terminalis r | 165 | 196 | 116 |
| fornix stria terminalis l | 260 | 245 | 150 |
| superior longitudinal fasciculus r | 424 | 1017 | 1054 |
| superior longitudinal fasciculus l | 684 | 1131 | 1009 |
| superior fronto-occipital fasciculus r | 81 | 96 | 71 |
| superior fronto-occipital fasciculus l | 77 | 81 | 63 |
| uncinate fasciculus r | 0 | 27 | 0 |
| uncinate fasciculus l | 5 | 28 | 19 |
| tapetum r | 40 | 40 | 35 |
| middle cerebellar peduncle | 1107 | 1498 | 275 |

**Supplementary Table 2.** TBSS results. Number of thresholded voxels per tract for the overall contrast (patients < controls) for the FA, MD and RD metrics.

| **TOI** | **FA** | **AD** | **MO** |
| --- | --- | --- | --- |
| middle cerebellar peduncle | 39 | 1 | 0 |
| pontine crossing tract | 3 | 0 | 0 |
| genu of corpus callosum | 1051 | 779 | 685 |
| body of corpus callosum | 1683 | 1836 | 2132 |
| splenium of corpus callosum | 4 | 2370 | 1820 |
| corticospinal tract r | 80 | 46 | 0 |
| corticospinal tract l | 131 | 12 | 0 |
| cerebral peduncle r | 246 | 190 | 0 |
| cerebral peduncle l | 321 | 283 | 0 |
| anterior limb of internal capsule r | 520 | 419 | 0 |
| anterior limb of internal capsule l | 441 | 311 | 0 |
| posterior limb of internal capsule r | 799 | 655 | 0 |
| posterior limb of internal capsule l | 815 | 690 | 37 |
| retrolenticular part of internal capsule r | 236 | 293 | 0 |
| retrolenticular part of internal capsule l | 221 | 424 | 596 |
| anterior corona radiata r | 683 | 345 | 186 |
| anterior corona radiata l | 643 | 24 | 7 |
| superior corona radiata r | 1125 | 385 | 11 |
| superior corona radiata l | 925 | 135 | 5 |
| posterior corona radiata r | 170 | 251 | 26 |
| posterior corona radiata l | 125 | 211 | 156 |
| posterior thalamic radiation r | 109 | 231 | 0 |
| posterior thalamic radiation l | 225 | 410 | 535 |
| sagittal stratum r | 2 | 25 | 0 |
| sagittal stratum l | 184 | 297 | 157 |
| external capsule r | 163 | 65 | 0 |
| external capsule l | 303 | 56 | 80 |
| cingulum cingulate gyrus r | 132 | 126 | 0 |
| cingulum cingulate gyrus l | 0 | 10 | 345 |
| fornix stria terminalis r | 91 | 0 | 0 |
| fornix stria terminalis l | 193 | 167 | 187 |
| superior longitudinal fasciculus r | 506 | 265 | 0 |
| superior longitudinal fasciculus l | 504 | 0 | 4 |
| superior fronto-occipital fasciculus r | 65 | 56 | 0 |
| superior fronto-occipital fasciculus l | 38 | 13 | 0 |
| uncinate fasciculus r | 7 | 0 | 0 |
| uncinate fasciculus l | 1 | 0 | 0 |

**Supplementary Table 3.** Number of thresholded voxels per tract for the Savings contrast (patients slope > controls slope) for the FA, AD, and MO metrics.

| **TOI** | **FA** |
| --- | --- |
| middle cerebellar peduncle | 913 |
| pontine crossing tract | 9 |
| genu of corpus callosum | 622 |
| body of corpus callosum | 1922 |
| splenium of corpus callosum | 1721 |
| corticospinal tract r | 138 |
| corticospinal tract l | 32 |
| medial lemniscus r | 91 |
| medial lemniscus l | 134 |
| inferior cerebellar peduncle r | 160 |
| inferior cerebellar peduncle l | 76 |
| superior cerebellar peduncle r | 213 |
| superior cerebellar peduncle l | 100 |
| cerebral peduncle r | 364 |
| cerebral peduncle l | 374 |
| anterior limb of internal capsule r | 413 |
| anterior limb of internal capsule l | 428 |
| posterior limb of internal capsule r | 765 |
| posterior limb of internal capsule l | 777 |
| retrolenticular part of internal capsule r | 495 |
| retrolenticular part of internal capsule l | 410 |
| anterior corona radiata r | 682 |
| anterior corona radiata l | 728 |
| superior corona radiata r | 612 |
| superior corona radiata l | 677 |
| posterior corona radiata r | 438 |
| posterior corona radiata l | 445 |
| posterior thalamic radiation r | 827 |
| posterior thalamic radiation l | 760 |
| sagittal stratum r | 369 |
| sagittal stratum l | 183 |
| external capsule r | 490 |
| external capsule l | 427 |
| cingulum cingulate gyrus r | 49 |
| cingulum cingulate gyrus l | 211 |
| fornix stria terminalis r | 132 |
| fornix stria terminalis l | 182 |
| superior longitudinal fasciculus r | 231 |
| superior longitudinal fasciculus l | 460 |
| superior fronto-occipital fasciculus r | 39 |
| superior fronto-occipital fasciculus l | 38 |
| uncinate fasciculus r | 17 |
| uncinate fasciculus l | 4 |
| tapetum r | 39 |
| tapetum l | 3 |

**Supplementary Table 4.** Number of thresholded voxels per tract for the contrast testing the correlation between FA and performance during the motor learning experiment. Threshold was set to < 0.09.
